# Emergence of Novel Coronavirus 2019-nCoV: Need for Rapid Vaccine and Biologics Development

**DOI:** 10.3390/pathogens9020148

**Published:** 2020-02-22

**Authors:** Balamurugan Shanmugaraj, Ashwini Malla, Waranyoo Phoolcharoen

**Affiliations:** 1 Research unit for Plant-produced Pharmaceuticals, Chulalongkorn University, Bangkok 10330, Thailand; balasbm17@gmail.com (B.S.); malla164_ashwini@yahoo.co.in (A.M.); 2 Department of Pharmacognosy and Pharmaceutical Botany, Chulalongkorn University, Bangkok 10330, Thailand

**Keywords:** biopharmaceuticals, coronavirus, emerging threat, infectious diseases, plant system, transient expression, viruses, zoonoses

## Abstract

Novel Coronavirus (2019-nCoV) is an emerging pathogen that was first identified in Wuhan, China in late December 2019. This virus is responsible for the ongoing outbreak that causes severe respiratory illness and pneumonia-like infection in humans. Due to the increasing number of cases in China and outside China, the WHO declared coronavirus as a global health emergency. Nearly 35,000 cases were reported and at least 24 other countries or territories have reported coronavirus cases as early on as February. Inter-human transmission was reported in a few countries, including the United States. Neither an effective anti-viral nor a vaccine is currently available to treat this infection. As the virus is a newly emerging pathogen, many questions remain unanswered regarding the virus’s reservoirs, pathogenesis, transmissibility, and much more is unknown. The collaborative efforts of researchers are needed to fill the knowledge gaps about this new virus, to develop the proper diagnostic tools, and effective treatment to combat this infection. Recent advancements in plant biotechnology proved that plants have the ability to produce vaccines or biopharmaceuticals rapidly in a short time. In this review, the outbreak of 2019-nCoV in China, the need for rapid vaccine development, and the potential of a plant system for biopharmaceutical development are discussed.

## 1. Introduction

The global health system consists of a network of organizations, including many private and public health sectors operating at different regional or global levels that have developed a stringent system that can provide effective protection to humans against emerging and re-emerging diseases. Though mortality associated with various infectious diseases have reduced in recent years and global life expectancy has increased in many parts of the world, infectious disease threats still remain one of the major global challenges and concerns even now [1]. The global health system is often confronted by emerging pathogens responsible for expanding an array of infectious diseases such as Zika, Chikungunya, Ebola, Nipah, Severe Acute Respiratory Syndrome (SARS), Middle East Respiratory Syndrome (MERS), and Influenza. The emergence of the 2019 novel coronavirus (2019-nCoV) has recently added to the list of problematic emerging pathogens in the 21st century, which was suspected to originate from the persons exposed to a seafood or wet market in Wuhan, Hubei Province, China, suggesting animal-to-human transmission [2,3]. This virus strain is previously unknown and was reported to infect humans for the first time. The virus continues to expand rapidly throughout the world. Many confirmed and susceptible cases have been identified in Wuhan, China, and exported cases have also been reported in neighboring countries including Thailand, Japan, Korea, Taiwan, and other countries including the United States, Canada, and European countries, which proves that the virus has the potential for quick dissemination across borders. In response to the rapid spread of the virus, many countries have tightened their border security, investigating people showing symptoms, and have taken necessary emergency steps to control its spread. Due to the increasing number of cases in China and other countries, the WHO has declared the 2019-nCoV outbreak a global health emergency of international concern on 30 January 2020 [4].

## 2. Novel Coronavirus 2019-nCoV

Coronaviruses (CoVs) belongs to the family *Coronaviridae*, subfamily *Coronavirinae,* and the order Nidovirales. They are classified into four genera such as *Alphacoronavirus* and *Betacoronavirus,* both of which infect mammals, whereas *Gammacoronavirus* infect avian species, and *Deltacoronavirus* infect both mammalian and avian species. It is a large enveloped virus with a positive sense, single-stranded RNA genome of about 26 to 33 kb that is distributed broadly among birds, humans, and other mammals such as camels, bats, mice, dogs, and cats [5]. The genome is surrounded by a helical capsid and an envelope; the spike protein forms large protrusions in the envelope in the shape of a crown, which gives the virus a coronal appearance. The word ‘*corona*’ in Latin means crown [6,7].

Human coronaviruses (HCoVs) are a major group of coronaviruses that are known respiratory pathogens associated with respiratory and intestinal infections of varying severity, including the common cold, pneumonia, and bronchiolitis. Human coronaviruses such as hCoV-229E, OC43, NL63, and HKU1, usually cause mild infection in humans. However, the onset of *Betacoronaviruses*, severe acute respiratory syndrome coronavirus (SARS-CoV) outbreak in Guangdong Province, China in 2003, and the middle east respiratory syndrome coronavirus (MERS-CoV) disease outbreak in 2012 in the Middle East resulted in high pathogenicity in humans, which demonstrated the lethality of virus when they cross the species barrier from animals to humans. Both viruses are believed to be originated from bats and subsequently transmitted to humans [8]. HCoVs has evolved rapidly in recent years due to mutation, high nucleotide substitution rates, its ability to establish infection in a new host, and cross-species transmission [9].

In December 2019, China detected many cases of viral pneumonia-like disease similar to SARS that were confirmed to be caused by novel *Betacoronavirus*, provisionally called 2019 novel coronavirus (2019-nCoV). Since then, the novel coronavirus outbreak has raised attention throughout the world. Although the potential cause of the disease is still unknown, initial reports predicted that the virus is possibly of zoonotic origin. 2019-nCoV is the causative agent for severe respiratory infection in humans termed as novel coronavirus-infected pneumonia (NCIP) [10]. nCoV is the third known coronavirus that causes fatal respiratory diseases in humans after highly pathogenic viruses SARS-CoV and MERS-CoV. Chinese researchers isolated the novel coronavirus from the infected patient in early 2020. As the virus is closely related to other bat coronaviruses, it is suspected that the bats are the primary reservoir for the virus. However, it is still unclear that, if the virus transmitted to humans directly from the bats or whether through an intermediate host. Detailed understanding of the enzootic patterns of the virus, its evolution, and surveillance are essential to control the disease and possibly to prevent the future epidemics of similar viruses.

The explosive outbreak of 2019-nCoV in China has been spread rapidly in many countries, probably by human movement and travel. The geographical distribution of the virus has been increasing since the outbreak. In early February 2020, thousands of cases were confirmed in China and more than 300 cases were reported outside of China, and numbers of cases are escalating daily in all the provinces in China and other countries. The virus has transmitted rapidly and many cases were reported globally. As of February 8, 2020, nearly 34,598 affected cases and 723 fatalities were reported in China. Most of the confirmed cases were from the Wuhan city in Hubei province. Outside China, 288 other cases were confirmed globally, and one death was reported in Philippines. In Singapore, 33 cases were confirmed, 32 in Thailand, 25 in Japan, 24 in Korea, 15 each in Australia and Malaysia, 12 in United States, and some cases were reported in Germany, France, Vietnam, UAE, Canada, India, Philippines, Italy, UK, Russia, Nepal, Sri Lanka, Cambodia, Belgium, Finland, Sweden, and Spain as on early February [11]. Chinese health authorities, WHO, and most of the countries have responded fast, and immediate actions were taken to contain the virus. All the countries have implemented stringent border control, screening travelers for possible infection, and travel restrictions in order to prevent its spread. The rapid spread of this infection is frightening and also it causes both mortality and financial loss, which present the global concern of this emerging disease.

## 3. Transmission and Symptoms

The transmission of 2019-nCoV is often spread from person to person through the respiratory droplets generated during coughs or sneezes from an infected person. Human-to-human transmission is reported in countries such as Germany, Japan, Vietnam, and the United States [12]. The confirmed cases through inter-human transmission have increased the fear and panic accompanying the 2019-nCoV outbreak. It is still unknown whether the virus spreads only through human contact or if there is possible transmission through oral-fecal contact as well.

The incubation time varies from 2–14 days after infection. The clinical presentation of this infection resembles SARS-CoV characterized with fever, dry cough, and shortness of breath in most of the cases, whereas non-respiratory symptoms such as headache, muscle ache, dyspnoea, rhinorrhoea, sneezing, sore throat, diarrhea, nausea, and vomiting are also reported in few patients. The affected persons also develop acute respiratory distress syndrome. Cases with critical illness showed respiratory failure, septic shock, and organs failure, which require intensive care support [3,13,14].

At this time, the knowledge about this virus is limited. New cases and mortalities are increasing daily. As a newly emerging viral infection, there is no vaccine or anti-viral therapeutics to treat human coronavirus infection till now. As of now, preventing infection is the current priority for disease control. The current protocol for infected patients is to quarantine and provide supportive management and palliative care. The best way to avoid the virus infection is to keep oneself away from infected people and the utmost personal hygienic care is essential. Quarantine measures shall be taken to separate, restrict the movement of infected people, and also the normal population from the regions where there is an epidemic outbreak. The WHO recommended precautionary measures to the general public, such as frequently cleaning hands, wearing a face mask, avoiding close contact with the infected persons or farm animals, and avoiding consumption of raw or half-cooked meat/eggs and following good food safety practices [11].

## 4. Perspectives on Biopharmaceuticals Development

There is an urgent need to develop rapid diagnostic tools and vaccines or post-exposure prophylaxis to treat this infection. Reliable, timely laboratory diagnosis and an effective vaccine are crucial for effective disease management and public health intervention. An effective vaccine should be affordable, and also the production platform should produce suitable vaccine candidates rapidly at low cost, especially during a disease outbreak. The advantages and disadvantages of the current expression systems for recombinant protein production are given in Table 1. Currently, plant expression system offers many advantages over other conventional systems that have the potential to tackle the production of vaccine candidates rapidly at affordable cost facilitating the global vaccination programs, especially in resource-poor nations where the vaccines are needed most [15].

In recent years, plants are being focused more on the production of biopharmaceuticals and virus-like particles (VLP’s). The technologies employed for the production of plant-made vaccines are stable nuclear expression, transient expression, and chloroplast expression. Based on several vital factors like yield, quality, time, and cost, appropriate technology can be chosen for producing biopharmaceuticals. Recent advancements in the plant expression strategies, especially the development of transient expression system or viral vectors, resulted in a huge increase in the protein yield that makes this plant host system, a promising system for the production of various biopharmaceutical proteins [16]. Several reports in the last two decades have enough evidence to prove that the plant produced biopharmaceuticals are as effective as the mammalian cell-based proteins and also elicit potent neutralizing antibodies, or shown therapeutic effects against the particular pathogen or infection [17,18,19].

The use of plants for the production of recombinant proteins and biopharmaceuticals has been gaining importance since the plant produced biologic taliglucerase alfa has been commercialized in 2012 against Gaucher’s disease that proclaimed a new era for plant made biopharmaceutical and triggered the innovation in the field of biopharmaceuticals [20]. Furthermore, many plant-produced candidates are in the clinical pipeline close to commercialization. Some of the examples of plant-produced recombinant antigens and monoclonal antibodies for infectious diseases are given in Table 2. Currently, countries, including Thailand, India, Japan, Korea, and the European community, are majorly involved in developing plant biopharmaceuticals against several human diseases [21]. Many reports reviewed the importance of plant expression system for the rapid production of candidate vaccines and therapeutic antibodies against infectious diseases [22,23,24,25,26,27].

Many biopharmaceutical companies have shifted their momentum towards the plant expression system by knowing its importance. Various plants such as tobacco, duckweed, moss, alfalfa, and other plants have been used for the production of plant-made pharmaceuticals. In September 2015, Leaf Bio, Inc., the commercial partner of Mapp Biopharmaceutical, Inc. (Mapp), received fast track designation by the U.S FDA for their plant-made biopharmaceutical called ZMapp for the treatment of Ebola virus disease. The authorization of plant-based biopharmaceutical ZMapp for emergency use in the earlier EBV outbreak by FDA is potentially a major breakthrough in the plant molecular farming field, as it serves as an endorsement of the technology to potential investors and grant funding agencies [50,55,56]. Many vaccine antigens expressed in plants are in clinical or advanced pre-clinical trials. Major players in the global plant-based biologics market include PlantForm, IBio Inc., Mapp Biopharmaceutical, Inc., Pfizer Inc., Ventria Bioscience, Medicago Inc., Greenovation Biotech GmbH, Kentucky BioProcessing, PhycoBiologics Inc., Synthon, Fraunhofer IME, Healthgen, Planet Biotechnology, and Icon Genetics GmbH.

As plant-made biopharmaceuticals provide efficacious and cost-effective strategies to protect against emerging infectious diseases, plant expression systems can be employed for the development of vaccines against nCoV. Transient expression in plants can be adapted for biopharmaceutical protein production when it is necessary to produce ‘rapid response vaccines’ as it produces more protein in a short time. The plant-based biopharmaceutical production against 2019-nCoV will include the identification of potential epitopes and production of full-length viral surface proteins present in the envelope region or production of subunit vaccines expressing immunogenic region or chimeric proteins.

The receptor-binding domain (RBD) in spike protein located on the outer membrane of coronavirus mediates receptor binding and plays a major role in virus entry into the host cell [57]. This protein could be used as a potential vaccine candidate as it is the major target for neutralizing antibodies [58]. Hence, it could be considered to develop potential effective vaccines or therapeutics against coronavirus infection. As the virus uses angiotensin-converting enzyme 2 (ACE2), the host cell receptor for its cell entry similar like SARS-CoV [59,60,61], the monoclonal antibodies that are identified and tested to be effective against SARS virus protein or specific to ACE2 can be produced in plants and shall be tested for its efficacy against nCoV. Earlier reports showed several vaccines and monoclonal antibody candidates in response to SARS-CoV and MERS-CoV, which could be tested and used for passive immunotherapy for an immediate immune response [62,63,64]. The possibility of producing a VLP based vaccine is also feasible by expressing all the structural viral proteins that can assemble into VLPs in plants. The structural and functional studies of viral proteins in nCoV might help in designing better vaccines and specific therapeutics. Producing viral proteins in plants may further be helpful to evaluate their potential in developing diagnostic kits or the protection/therapeutic tools that can be manufactured fast in order to manage the highly infectious nCoV. This will open an avenue to characterize recombinant immunogenic viral proteins and provide a proof of concept for using plants as a robust, rapid, and flexible platform for producing protein/research reagents, which are highly essential to face potential nCoV outbreaks.

## 5. Concluding Remarks

The coronavirus outbreak has been declared a global health emergency and represents one of the greatest risks to global health, as the virus has a tendency to infect a large number of human populations, and the outbreak can cause severe medical complications with economic impact, particularly in middle-income countries where resources are limited for early diagnosis and preventive measures. Human mobility, air travel, and international trade can likely increase the number of cases in other regions as well. Continued surveillance along with the robust response of government agencies, medical practitioners, and researchers, is highly essential for the effective management of this emerging pathogen. Public health officials need to identify the source and virus reservoir, transmission cycle, pathogenesis, inter-human transmission, and clinical manifestations, which might be helpful to develop animal models, diagnostic reagents, anti-viral therapies, and vaccines against this pathogen. As the virus emerged suddenly and became a serious global concern, there is a need for rapid vaccine development. Although classical expression systems for biopharmaceutical proteins are still amenable, the development of transient expression in plants has deeply influenced the pharmaceutical sector to produce affordable vaccines and biologics rapidly at low cost. Hence, the plant expression platform shall be employed for biopharmaceutical production to accelerate the fight against this deadly infectious disease. The collaborative efforts of researchers are highly desirable to use a plant expression platform for producing an efficient cost-effective vaccine to control this epidemic. The continuous effort of research in this direction might be helpful in producing high-value biologics and pharmaceuticals on a large scale in a short time, especially during epidemics.

## Figures and Tables

**Table 1:** Advantages and disadvantages of different therapeutic protein production platforms.

Expression System	Advantages	Disadvantages
Bacteria	Easy to manipulate Low cost High expression Ease of scale up Short turnaround time Established regulatory procedures and approval	Improper folding Lack of post-translational modifications, which may affect the protein function. Endotoxin accumulation
Mammalian Cells	Proper folding and authentic post-translational modifications Existing regulatory approval	High production cost Expensive media and culture condition requirements
Yeast	Rapid growth and scalable Easy to manipulate Simple and inexpensive media requirements and culture conditions Post-translational modifications of recombinant proteins	Difficulty in cell disruption due to the thick and hard cell walls Hyperglycosylation of proteins
Insect cells	High expression levels Ability to produce complex proteins including secreted, membrane and intracellular proteins Proper folding and post-translational modifications	High cost and time consuming Expensive media and culture condition requirements
Plant	Rapid and affordable Optimized growth conditions Free from pathogen and bacterial toxin contaminants Economical Post-translational modification somewhat similar like mammalian system	Regulatory compliance Limited glycosylation capacity

**Table 2:** Examples of recombinant antigens and monoclonal antibodies expressed in plants.

Virus/Infection	Antigen/Protein	Plant host/Expression System
Vaccines		
Hepatitis B	Surface Antigen (HBsAg)	Potato [28] Lettuce [29] Maize [30]
Human Immunodeficiency Virus (HIV)	p24-Nef, C4V3, Multi-HIV	Tobacco [31,32,33]
	C4(V3)6 multi-epitopic protein	Lettuce [34]
	p24	*Arabidopsis* and Carrot [35]
Human papilloma virus (HPV)	HPV16-L1	Tobacco [36]
	HPVL1 VLPs	Tomato [37]
Influenza	H3N2 Nucleoprotein	Maize [38]
Rabies	G protein	Maize [39] Tomato [40]
Polio	VP1	Tobacco and Lettuce [41]
	PV3 VLPs	Tobacco [42]
Ebola	Ebola Immune Complex	Tobacco [43]
Antibodies		
Dengue	E60	Tobacco [44]
Human Immunodeficiency Virus (HIV)	VRC01, P2G12	Tobacco [45,46]
Rabies	E559	Tobacco [47]
	62-71-3	Tobacco [48]
	Single-chain antibody molecule (62-71-3)	Tobacco [49]
Ebola	ZMapp	Tobacco [50]
Influenza	KPF1-Antx	Tobacco [51]
Porcine epidemic diarrhea virus (PEDV)	2C10	Tobacco and Lettuce [52]
Chikungunya	CHKVmab	Tobacco [53]
West Nile Virus	pE16	Tobacco [54]

## Abbreviations

HCoVs	Human coronaviruses
MERS	Middle East Respiratory Syndrome
MERS-CoV	Middle East Respiratory Syndrome Coronavirus
nCoV	Novel Coronavirus
NCIP	Novel Coronavirus-Infected Pneumonia
SARS	Severe Acute Respiratory Syndrome
SARS-CoV	Severe Acute Respiratory Syndrome Coronavirus
FDA	Food and Drug Administration
VLP	Virus-like particles
WHO	World Health Organization
